# Catastrophic Collapse Can Occur without Early Warning: Examples of Silent Catastrophes in Structured Ecological Models

**DOI:** 10.1371/journal.pone.0062033

**Published:** 2013-04-11

**Authors:** Maarten C. Boerlijst, Thomas Oudman, André M. de Roos

**Affiliations:** Theoretical Ecology, Institute for Biodiversity and Ecosystem Dynamics, University of Amsterdam, Amsterdam, The Netherlands; Universitat Pompeu Fabra, Spain

## Abstract

Catastrophic and sudden collapses of ecosystems are sometimes preceded by early warning signals that potentially could be used to predict and prevent a forthcoming catastrophe. Universality of these early warning signals has been proposed, but no formal proof has been provided. Here, we show that in relatively simple ecological models the most commonly used early warning signals for a catastrophic collapse can be silent. We underpin the mathematical reason for this phenomenon, which involves the direction of the eigenvectors of the system. Our results demonstrate that claims on the universality of early warning signals are not correct, and that catastrophic collapses can occur without prior warning. In order to correctly predict a collapse and determine whether early warning signals precede the collapse, detailed knowledge of the mathematical structure of the approaching bifurcation is necessary. Unfortunately, such knowledge is often only obtained after the collapse has already occurred.

## Introduction

**Figure 1:**
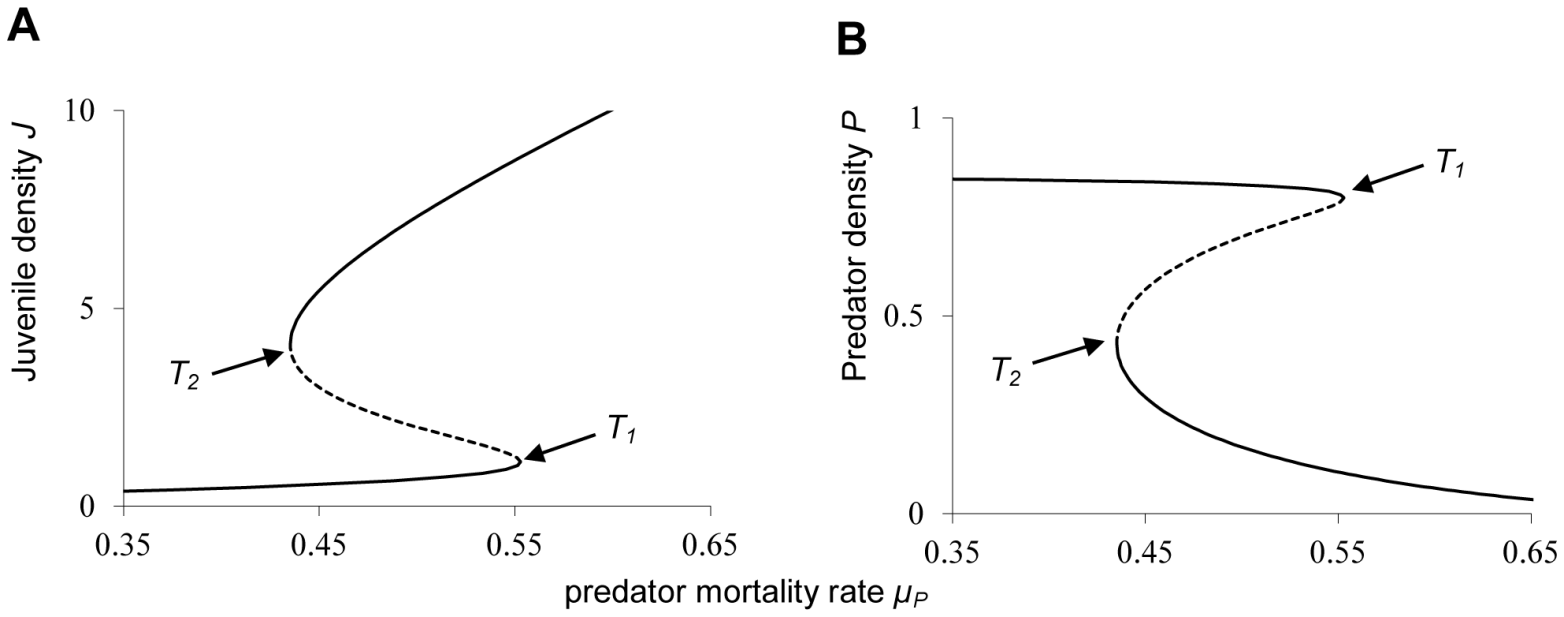
Bistability and catastrophic collapse in a structured predator-prey system. Bifurcation diagram as a function of predator death rate *µ_P_*. (**A**) Equilibrium juvenile density J, and (**B**) Equilibrium predator density P. The equilibrium curves exhibit a so-called catastrophe fold. Between the bifurcation points T1 (*µ_P_*≈0.553) and T2 (*µ_P_*≈0.435) the system is bistable (indicated by solid lines), with an intermediate saddle-node equilibrium (indicated by the dashed line) which is unstable. Model parameters are b = 1, c = 1, *µ_J_* = 0.05, *µ_A_* = 0.1.

Catastrophic regime shifts can occur in many ecosystems, and often such shifts cause substantial loss of biodiversity and ecosystem functioning [1]. Recently, the existence of generic early warning signals, which precede and can predict such a catastrophic collapse, has received much attention [2] [3] [4] [5] [6] [7], although the concept was already proposed much earlier [8]. Already in the seventies of the last century it was pointed out that catastrophic ecosystem collapses can be caused by an underlying fold bifurcation [9] [10]. Figure 1 shows such a so-called “catastrophe fold”, where, near the bifurcation points *T_1_* and *T_2_*, a slight change in the environmental conditions can cause a sudden shift from one stable state to another. As catastrophic shifts can cause substantial loss of biodiversity and ecosystem functioning, reliably predicting such catastrophes would be of crucial importance. Recently, it has been proposed that there exist generic early warning signs, based on a phenomenon that is coined “critical slowing down” near the bifurcation [6]. At the fold bifurcation, the eigenvalue of the dominant eigenvector becomes zero, and consequently, near the bifurcation, perturbations in the direction of this eigenvector only very slowly return to the equilibrium. As a result, the variance and autocorrelation will both steeply increase when approaching the fold bifurcation. This is a generic property of fold bifurcations, and it suggests that indeed generic early warning signals can be observed for natural ecosystems [7] [11] [12]. However, how robust and informative these early warning signals are for more complex ecosystems remains unclear.

## Methods

In this paper, we investigate a simple ecological model for the interaction between a predator and a stage-structured prey species. The model distinguishes between juvenile and adult prey individuals, and the predator only attacks adult prey. The model, in its most general form, consists of three ordinary differential equations for, respectively, juvenile (*J*), adult (*A*), and predator density (*P*): 
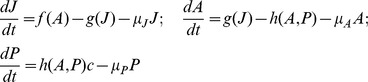



Here, *f*(*A*) is a function that specifies the reproduction rate of adults, *g*(*J*) specifies the maturation rate of juveniles, and *h*(*A,P*) is the predation rate on adults. Parameters *µ_J_*, *µ_A_* and *µ_P_* are death rates, and *c* is a conversion factor. We use a realization of this model with *f*(*A*) = *bA*, *g*(*J*)  = *J/(1+J^2^)*, and *h*(*A,P*)  = *AP*. For a description of the ecological setting of the model we refer to an earlier paper by van Kooten *et al.* [13]. Predation and fecundity are linear functions, and maturation is non-linear. In absence of predators the prey population is regulated by a maturation bottleneck at high juvenile density. Parameter *b* represents adult reproduction rate. Environmental noise is added to this model by randomly varying death rates every time unit, applying random (white), or correlated (pink) noise. This method of adding noise to the per capita death rates is giving similar results to directly perturbing population numbers after every time unit. For numerical integration we used the Cash-Karp Runge-Kutta method [14] for solving ordinary differential equations.

## Results

**Figure 2:**
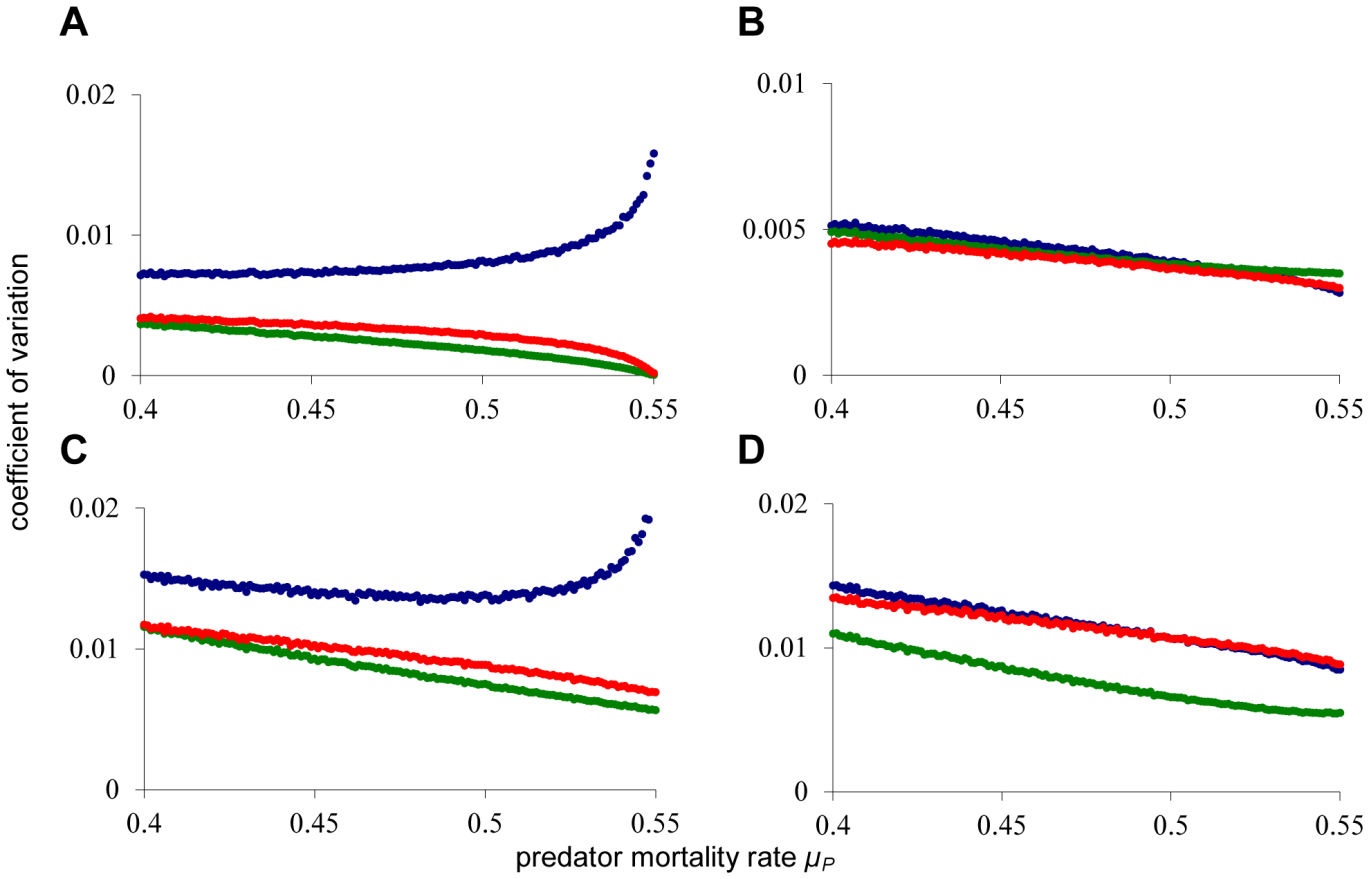
Early warning signals in coefficient of variation. For each value of *µ_P_*, starting with *µ_P_*  = 0.4, the model is simulated for 60,000 time units, of which the last 50,000 time units are used to compute population averages and variances. Hereafter, *µ_P_* is incremented with *Δµ_P_* = 0.001, towards the catastrophic collapse at *µ_P_*≈0.553. Death rates are perturbed every time unit using white noise with standard deviation σ = 0.005. (**A**) Noise added to the juvenile population (**B**) Noise added to the adult population (**C**). Independent noise added to all three populations. (**D**) Identical, fully correlated, noise added to all three populations. Colors are blue for juveniles, green for adults, and red for the predators. For other model parameters see Figure 1.

**Figure 3:**
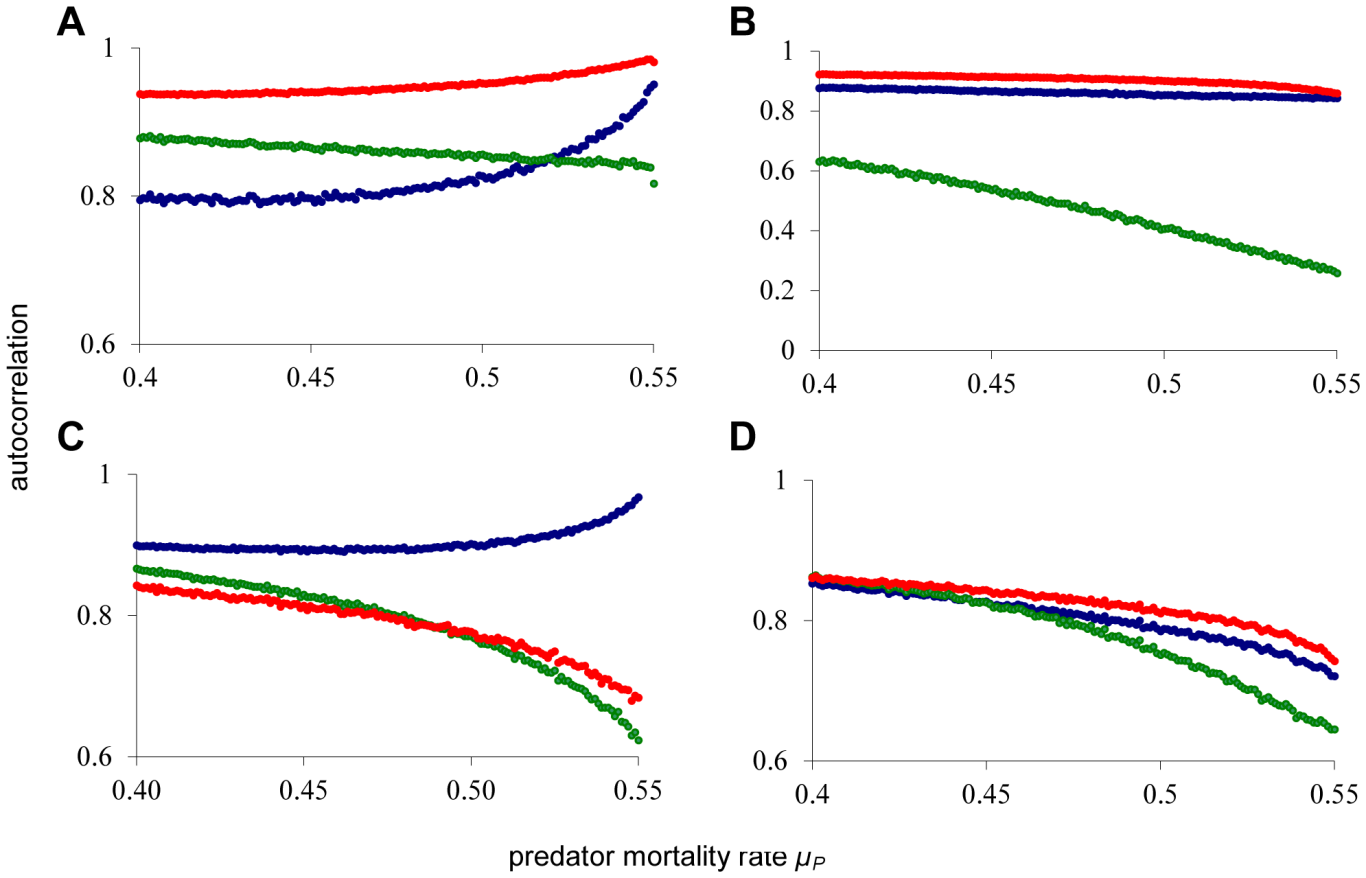
Early warning signals in autocorrelation. For the same simulation as in Figure 2, the lag-1 autocorrelation is computed over the last 50,000 time units. (**A**) Noise added to the death rate of the juvenile population (**B**). Noise added to the death rate of the adult population (**C**). Independent noise added to the death rates of all three populations. (**D**) Identical, fully correlated, noise added to the death rates of all three populations. For description of the simulation and for color index see Figure 2.

In Figures 2A through 2D, we gradually increase the predator death rate towards the catastrophic collapse of the predator density at *µ_P_*≈0.553 (corresponding to point *T_1_* in Figure 1), and we monitor the coefficient of variation for all three state variables. Notably, no early warning signal is observed for the variation in adult and predator density, regardless of the way in which environmental noise is added to the system. This is striking, because the predator is the species that will collapse at the catastrophe, and yet, it is not displaying any warning signal. There can be an early warning sign in the juvenile density, but only if noise is applied to the juvenile population (Figure 2A), or if noise is added independently to all three populations (Figure 2C). Most surprisingly, the catastrophic collapse happens without any prior warning if environmental noise is applied to the adult population (Figure 2B) or to all three populations in a correlated manner (Figure 2D). The latter procedure corresponds to a situation where changing environmental conditions affect all three populations equally. In Figures 3A through 3D it is shown that also autocorrelation does not provide reliable early warning signals for this system. In case of noise on the adult population (Figure 3B) and fully correlated noise (Figure 3D) all autocorrelations are decreasing towards the catastrophe. In case of noise on juveniles (Figure 3A) or independent noise to all variables (Figure 3C), a clear early warning signal again only occurs in the juvenile population.

**Figure 4:**
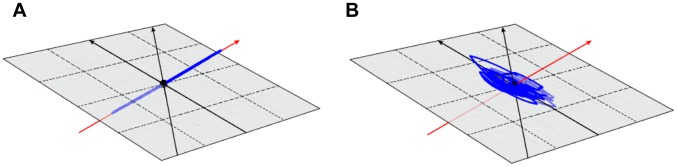
Effect of the direction of perturbation on early warning signals. Predator death rate is fixed at *µ_P_* = 0.55 (close to the catastrophe), and the death rate of either the juveniles or the adults is perturbed using white noise with standard deviation σ = 0.005. System trajectories are plotted in blue for 60 time units. The dominant eigenvector is indicated by the red arrow, and the second and third eigenvector are indicated by the black arrows. (**A**) When the juvenile death rate is perturbed, the system responds only in the direction of the dominant eigenvector, resulting in an early warning signal that is only apparent in juvenile population fluctuations. (**B**) When the adult death rate is perturbed, the system responds in the direction of the surface spanned by the second and third eigenvector (indicated in grey), resulting in damped oscillations and absence of early warning. For other model parameters see Figure 1. For an animated rotation of these 3D figures, and for direction and scaling of axis see Movie S1 and S2.

**Figure 5:**
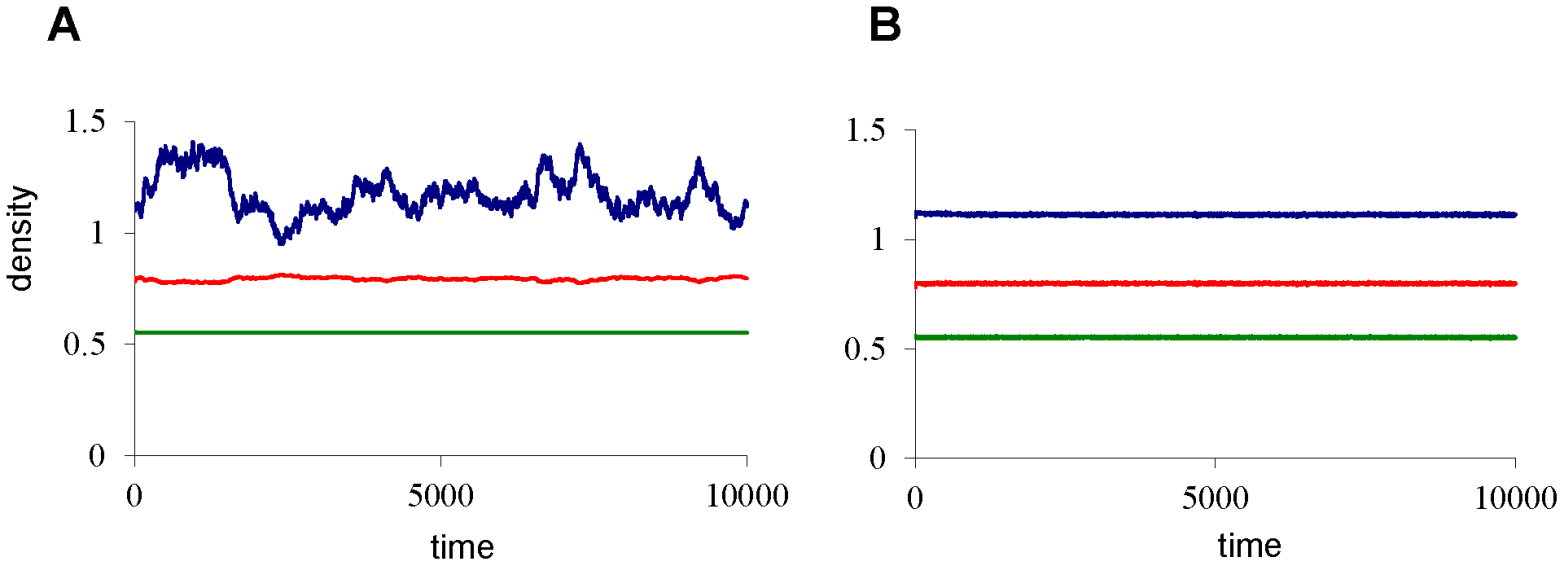
Early warning signals in the linearized system. The model of Figure 1 is linearized using the Jacobian matrix. Predator death rate is set at *µ_P_* = 0.5528 (very close to the bifurcation point T1 in Figure 1). The death rate of either the juveniles or the adults is perturbed using white noise with standard deviation σ = 0.005. (**A**) When noise is added to the juvenile death rate, the juvenile population (indicated in blue) clearly shows critically slowing down, whereas the adult (green line) and predator (red line) populations do not show early warning signs. (**B**) When noise is added to the adult death rate, all three populations do not show any sign of early warning. Note that the fluctuations in the juvenile population in Figure 5A are so large, that the full (i.c. not linearized) system would shift to the alternative steady state.

Apparently, the early warning signals are not generic at all, as a fold catastrophe can occur without prior warning. So, what is flawed in the argument that “critical slowing down” is a generic property of all fold bifurcations? In fact, the statement is completely correct, but the slowing down only occurs in the direction of the dominant eigenvector. In our system, this eigenvector, near the bifurcation, almost exclusively points in the direction of the axis corresponding to the juvenile population. Figure 4A shows that adding noise to the juvenile population will result in the system responding only in the direction of this dominant eigenvector, causing the early warning signal in the juvenile density. In contrast, in Figure 4B, when noise is added to the adult population, perturbations mainly develop in the direction of the second and third eigenvector. The eigenvalues pertaining to these eigenvectors are imaginary, and their real parts become more negative towards the catastrophe, causing increasingly damped oscillations and absence of critical slowing down. As a consequence, the forthcoming collapse is camouflaged. We also examined the linear stability of the system by using the Jacobian matrix (see [7]). Adding noise to the linearized system gives a very similar early warning signature in approaching the bifurcation. In Figure 5A and 5B the linearized system is brought very close to the bifurcation (at *µ_P_* = 0.5528). In Figure 5A, where noise is added to the juvenile population, this population is clearly showing critically slowing down, with very large and slow excursions from the equilibrium, whereas the adult and predator population do not show an early warning signal. In contrast, in Figure 5B, where the same amount of noise is added to the adult population, early warning signals are completely absent from all three populations.

**Figure 6:**
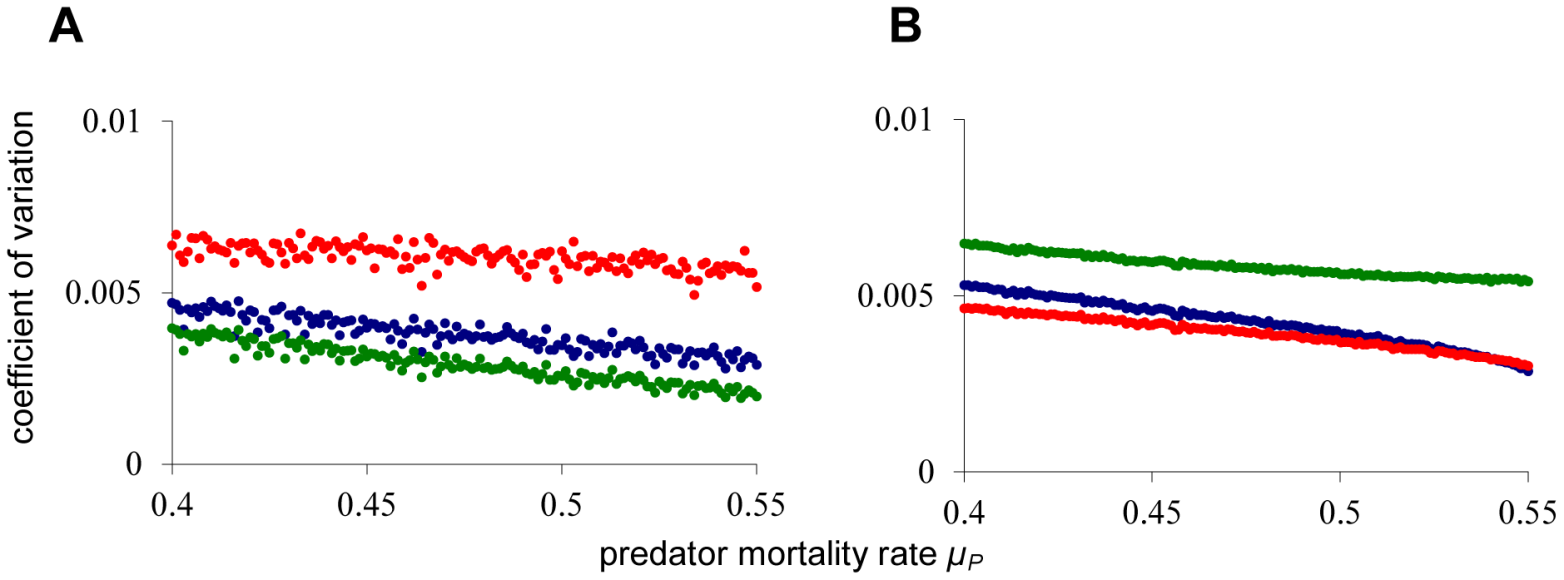
Early warning signals with correlated noise and discrete noise. Coefficient of variation and autocorrelation are monitored for increasing predator death rate towards the catastrophic collapse at *µ_P_*≈0.553. Bifurcation procedure, parameters and colors are identical to Figure 2. (**A**) Coefficient of variation when pink noise (1/f correlated noise) is added to the death rate of the adult population. (**B**) Coefficient of variation when discrete white noise is applied directly to the adult population numbers after each time step.

**Figure 7:**
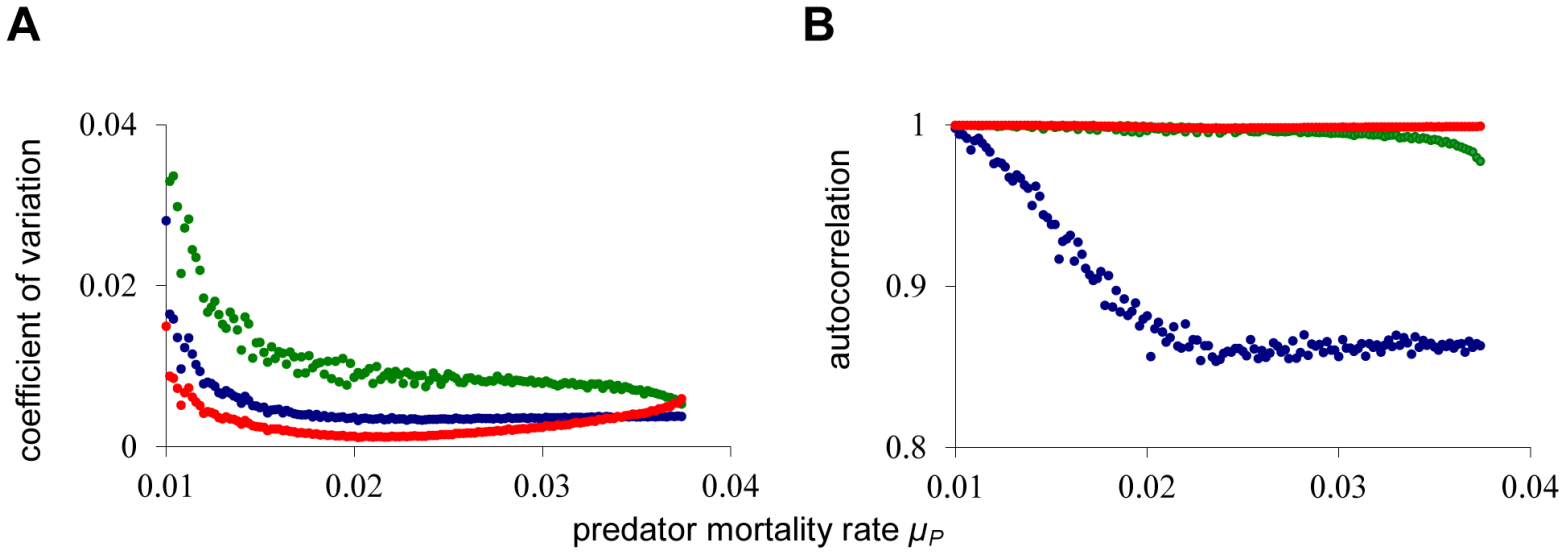
Early warning signals in the fully size-structured population model of de Roos and Persson [**17**]. Independent white noise with σ = 0.002 is added to the death rates of all juvenile consumers. Bifurcation procedure and colors are identical to Figure 2, with predator mortality staring at *µ_P_* = 0.01 (note that the original article uses parameter δ instead of *µ_P_*), and incremented with *Δµ_P_* = 0.0002 after each 50,000 time units. The fold catastrophe in this model is located at approximately *µ_P_* = 0.038. Coefficient of variation and lag-1 autocorrelation are computed for each value of *µ_P_* over the last 40,000 time steps. (**A**) Coefficient of variation, and (**B**) Autocorrelation. All other parameters have default values as used by de Roos and Persson [17].

We extensively tested robustness of the silent collapse phenomenon, e.g. by varying the type of noise. In Figure 6A, more natural correlated ‘pink’ noise [15] is added to the death rate of the adult population (using the method of Vasseur and Yodzis [16]). The correlated noise is increasing the system variance, but the approach to the catastrophe remains completely silent. In Fig 6B, discrete white noise is, after each time unit, directly applied to the adult population numbers. Also with this type of noise the approach to the catastrophe is not showing any early warning signals. We furthermore investigated the presence of early warning signals near the second bifurcation point in our system (i.c. *T_2_* in Figure 1). Also in this case adding noise on the adult population results in a silent approach to the catastrophe. Finally, we have investigated the presence of early warning signals in a much more complex, fully size-structured population model of de Roos and Persson [17]. This model consists of three trophic levels, and the consumer population is structured according to individual body size. White noise is applied to the death rate of all juvenile consumers, independently varying every time unit, and the predator mortality rate is slowly increased towards the catastrophic collapse at µ*_P_*≈0.038. In Figure 7A and 7B, it can be seen that also in this system early warning for the catastrophe is absent from the juvenile and adult consumer populations, irrespective of whether the coefficients of variation or the autocorrelations are considered.

## Discussion

We have shown three examples where catastrophic collapse can occur without prior early warning signals in autocorrelation or variance. Although critical slowing down is a universal property of fold bifurcations, this does not mean that the increased sensitivity will necessarily manifest itself in the system variables. Instead, whether the population numbers will display early warning will depend on the direction of the dominant eigenvector of the system, that is, the direction in which the system is destabilizing. This theoretical point also applies to other proposed early warning signals, such as skewness [18], spatial correlation [19], and conditional heteroscedasticity [20]. In our main example, early warning signal only occurs in the juvenile population, which in fact could easily be overlooked in ecological systems (e.g. exploited, marine fish stocks), as often only densities of older, more mature individuals are monitored. Furthermore, the early warning signals can in some cases be completely absent, depending on the direction of the perturbations to the system. In our paradigm system, the other two eigenvectors induce increasing stability near the fold bifurcation, thus obscuring the early warning signal. This mechanism is reminiscent of so-called “chaos control” [21], where perturbation towards a stable manifold can act to stabilize system dynamics. We have demonstrated that our results also apply to a fully size-structured population model.

We want to stress that we have used ecological models that have been proposed before [13] [16], and that have been shown to connect to experimental ecological data [22]. The fact that we have found three silent catastrophes in published ecological models indicates that the phenomenon is not extremely rare. However, how generic these results are for other ecological systems remains to be investigated. In general, when systems become more complex it is to be expected that early warning signals become less pronounced. The reason for this is that with an increasing number of variables, the number of eigenvectors also increases, and consequently the system is less dominated by the critical slowing down along the dominant eigenvector.

We have focused on catastrophic regime shifts that are caused by an underlying fold bifurcation, as this cause of bistability has received most attention in the ecological literature. However, catastrophic shifts can also be induced by other underlying mathematical structures, such as a subcritical Hopf bifurcation or a bifurcation to a chaotic attractor. For this latter possibility it has recently been demonstrated that also in this case early warning signals can be absent [23]. Furthermore, early warning can be weak if environmental noise is coupled to a system parameter that has a decreasing contribution to the dynamics when approaching the catastrophe [24]. To even further limit the predictive value of early warning signals, these signals can also occur in approaching other, non-catastropic, bifurcations, such as a Hopf or a transcritical bifurcation [25]. Also, transient dynamics resulting from e.g. changing environmental conditions can generate early warning signals without a forthcoming collapse. In conclusion, we propose to reject the currently popular hypothesis that catastrophic shifts are preceded by universal early warning signals. We have provided counterexamples of silent catastrophes, and we have pointed out the underlying mathematical reason for the absence of early warning signals. In order to assess whether specific early warning signals will occur in a particular system, detailed knowledge of the underlying mathematical structure is +necessary. Using early warning signals as a tool for nature conservation could still proof to be a good general policy, but it definitely needs more theoretical underpinning.

## Supporting Information

Movie S1 **Animated rotation of** **Figure 4A** **.** White noise is added to the juvenile prey death rate, which induces excursions of the system state in the direction of the dominant eigenvector (indicated by the red arrow). As a result, the system only responds along this eigenvector (trajectory in blue), leading to an early warning signal that only appears in the juvenile population.
(MOV)

Movie S2 **Animated rotation of** **Figure 4B** **.** White noise is added to the adult prey death rate, which induces excursions of the system state in the direction of the surface (indicated in grey) corresponding to the second and third eigenvector (black arrows). As a result, the system responds with damped oscillations (trajectory in blue), leading to a total absence of early warning signals.
(MOV)
